# Still Wanting to Win: Reward System Stability in Healthy Aging

**DOI:** 10.3389/fnagi.2022.863580

**Published:** 2022-05-30

**Authors:** Laura Opitz, Franziska Wagner, Jenny Rogenz, Johanna Maas, Alexander Schmidt, Stefan Brodoehl, Carsten M. Klingner

**Affiliations:** ^1^Hans Berger Department of Neurology, Jena University Hospital, Jena, Germany; ^2^Biomagnetic Center, Jena University Hospital, Jena, Germany; ^3^Clinician Scientist Program OrganAge, Jena University Hospital, Jena, Germany

**Keywords:** healthy aging, EEG, reward, functional connectivity, HAROLD, PASA

## Abstract

Healthy aging is accompanied by multi-faceted changes. Especially within the brain, healthy aging exerts substantial impetus on core parts of cognitive and motivational networks. Rewards comprise basic needs, such as food, sleep, and social contact. Thus, a functionally intact reward system remains indispensable for elderly people to cope with everyday life and adapt to their changing environment. Research shows that reward system function is better preserved in the elderly than most cognitive functions. To investigate the compensatory mechanisms providing reward system stability in aging, we employed a well-established reward paradigm (Monetary Incentive Delay Task) in groups of young and old participants while undergoing EEG measurement. As a new approach, we applied EEG connectivity analyses to assess cortical reward-related network connectivity. At the behavioral level, our results confirm that the function of the reward system is preserved in old age. The mechanisms identified for maintaining reward system function in old age do not fit into previously described models of cognitive aging. Overall, older adults exhibit lower reward-related connectivity modulation, higher reliance on posterior and right-lateralized brain areas than younger adults, and connectivity modulation in the opposite direction than younger adults, with usually greater connectivity during non-reward compared to reward conditions. We believe that the reward system has unique compensatory mechanisms distinct from other cognitive functions, probably due to its etymologically very early origin. In summary, this study provides important new insights into cortical reward network connectivity in healthy aging.

## Introduction

In a globally aging society, understanding motivational and cognitive processes in the elderly are of growing importance. A functionally intact reward system remains indispensable in healthy aging to succeed in daily life. Reward prediction and receiving are involved in every part of an individual’s behavior, shaping its actions to obtain rewards and avoid punishment (Schultz, 1998; Wise, 2004; Berridge and Kringelbach, 2008). Reward measurably improves cognitive performance and decision-making in humans (Spaniol et al., 2014; Cohen et al., 2016; Cox and Witten, 2019; Bowen et al., 2020). Cognition is inseparably intertwined with reward processing (Pessoa and Engelmann, 2010). Rewards exert extensive influences on behavior, mediating multi-faceted cognitive processes (Braver et al., 2014). In this way, reward-based enhancement of cognitive performance is conveyed through cognitive control processes (Jimura et al., 2010; Pessoa and Engelmann, 2010). Cognitive as well as reward functions critically rely on cortical areas like the PFC and parietal cortex, serving the integration of reward and cognitive control networks (Pessoa and Engelmann, 2010; Braver et al., 2014; Chau et al., 2018). Across these links, the reward network functionally interacts with cognition-related large-scale networks like the frontoparietal control network (FPCN), default network (DN), and ventral and dorsal attention network (VAN, DAN) (Raichle et al., 2001; Corbetta and Shulman, 2002; Vincent et al., 2008; Spaniol et al., 2015; Parro et al., 2018). The reward system’s core part is the mesocorticolimbic dopamine system, comprising projections of midbrain dopamine neurons to cortical structures, such as the medial prefrontal (mPFC) and orbitofrontal cortex (OFC) (Wise, 2004; Björklund and Dunnett, 2007; Lammel et al., 2008; Haber and Knutson, 2010; Ikemoto, 2010).

All the structures and mechanisms involved are subject to age-related changes. For instance, brain volume decreases with age up to 0.5% per year at the age of 60 (Hedman et al., 2012); locally pronounced in prefrontal areas (Gordon et al., 2008; Raz et al., 2010; Zanto and Gazzaley, 2019). Furthermore, the most important neurotransmitter in the reward system, the dopamine system (Wise, 2002; Schultz, 2007) declines with age (Fearnley and Lees, 1991; Backman et al., 2006; Li et al., 2009; Karrer et al., 2017).

These multilayered structural changes inevitably affect related brain functions, not only in the reward system but also in a wide range of cognitive areas. Interestingly, not all cognitive domains are equally affected by age-related changes (Lyoo and Yoon, 2017).

Recent studies have indicated that the reward system’s function is less affected by age-related changes than other highly interconnected functions like fluid cognitive domains (Blum et al., 2018; Tryon et al., 2020). Reward sensitivity remains preserved in age as older people are able to exhibit enhanced performance (Spaniol et al., 2015; Castel et al., 2016; Yee et al., 2019) and restoration of age-impaired cognitive abilities under incentive motivation (Ferdinand and Czernochowski, 2018). With respect to this divergency, however, it remains an open question how the aging brain manages to preserve the functionality of the reward system despite the cellular and molecular changes affecting its core parts. In general, older adults attempt to preserve their cognitive abilities with reduced resources by employing compensatory mechanisms. These compensatory mechanisms are well-observed in fMRI brain network alterations in older adults that comprise hypo- and hyperactivations in specific patterns that have been summarized in several models. Three of these models characterizing mechanisms of cognitive aging are the HAROLD (Hemispheric Asymmetry Reduction in Older Adults) (Cabeza, 2002), the PASA (posterior-anterior shift in aging) model (Davis et al., 2008), and the theory of frontoparietal control network (FPCN) hyperactivation (Reuter-Lorenz and Park, 2014; Li et al., 2015). The HAROLD model describes bilateral frontal cortical activation in older adults in comparison to lateralized frontal cortical activation in younger adults (Cabeza, 2002; Cabeza et al., 2018). PASA coherently describes a prefrontal over-related to an occipital underrecruitment in older compared to younger adults (Davis et al., 2008). FPCN network hyperactivation, HAROLD, and PASA all have been found to be associated with improved cognitive performance in healthy aging (Li et al., 2015). Qualifying that, HAROLD and PASA or FPCN hyperactivation to date have been tested using demanding cognitive tasks. Additionally, these models have been proposed to reflect aging mechanisms in salience networks (Jacques et al., 2013). Compensatory reorganization of cognitive networks during reward processing has already been reported in healthy aging (Spaniol et al., 2015). It remains unclear which compensatory mechanisms underlie reward system function in healthy aging and whether these models are able to explain preserved reward system stability.

We performed a study contrasting older and younger participant groups in a monetary incentive delay task (MID) using electroencephalography (EEG) (Knutson et al., 2000). Reward-guided acting can be separated into two distinct temporal phases, the prediction and the receiving phases. The MID allows to dissociate both phases. In the prediction phase, a reward-predicting stimulus elicits specific approach behavior, serving the obtaining of a reward (Schultz, 2006). Reward receipt is accompanied by pleasure, known as hedonia (Berridge and Kringelbach, 2015; Becker et al., 2019). EEG offers a high temporal resolution and, therefore, provides detailed information on the time course of neuronal information processing (Andrä and Nowak, 2007). Rapidly changing network states can be investigated by employing functional connectivity analyses (Friston, 2011; Fries, 2015; Meyer et al., 2021).

Studies investigating reward processing in healthy aging largely confirmed the notion of preserved reward sensitivity in older adults. In EEG studies, reward prediction has been related to increased frontal theta power (Doñamayor et al., 2012; Gruber et al., 2013), whereas reward consumption was associated with high-beta oscillations (20-35 Hz) in younger adults (Marco-Pallarés et al., 2015). Only one study investigated age-related effects of incentives on brain oscillations, reporting increased frontocentral and parietooccipital theta power for reward anticipation in older but not younger adults (Steiger and Bunzeck, 2017). To date, no EEG studies investigating aging effects on the reward system with complex functional connectivity analyses, especially in the context of compensation, have been published (Meyer et al., 2021).

This study aims to further understand the neural mechanisms providing for generally stable reward system function in the elderly. Therefore, we first hypothesized a behaviorally preserved reward system function in older adults. For investigating reward-based modulation of large-scale brain networks, functional brain connectivity analyses were employed. Based on previous studies, diminished neuronal reward effects on older adults are expected. We hypothesized altered responses to occur in reward-related frequency bands: in the alpha band due to its involvement in attentional processes (Sadaghiani and Kleinschmidt, 2016) and reward prediction (Heuer et al., 2017); in the delta band as it has been associated with reward, motivation, and salience detection (Knyazev, 2012); in the theta and high-beta bands for their roles in reward feedback processing (Luft, 2014; Andreou et al., 2017; Glazer et al., 2018). Furthermore, we especially focused on compensatory mechanisms within the reward system and changes in cognition-related networks during reward processing in older adults. Due to the close link between reward processing and cognition, we propose established functional compensatory mechanisms of cognitive control (i.e., HAROLD, PASA, and FPCN hyperactivation) to apply to preserved reward system function in healthy aging.

## Materials and Methods

### Participants

This study was comprised of 46 healthy volunteers (Table 1) with no history of neurological or psychiatric disease, who were divided into 2 groups according to their age. The younger group comprised 22 subjects (13 women), aged 18 to 33 years (mean, 24.59 ± 3.96), and the older group, 24 subjects (13 women), aged 62 to 86 years (mean, 69.42 ± 5.85). Prior to the monetary incentive delay task (MID), the subjects had undergone an assessment with an anamnesis questionnaire and standardized questionnaires (Edinburgh Handedness Inventory, BDI-II, SF-36 (Kirchberger, 2000), EQ-5D (EuroQol Group, 1990), and the MoCA (Nasreddine et al., 2005) test for the older group (Table 1). All the subjects were right-handed according to the Edinburgh Handedness Inventory (Oldfield, 1971). We explicitly tested for the presence of depressive symptoms and included only the subjects with a BDI-II Score < = 14 points (Beck et al., 1996). Further exclusion criteria were sight defects, substance dependence, and current ingestion of psychopharmaca. From the initial sample of 49 subjects, data of three subjects had to be excluded from analysis because of red-green color blindness (one subject), technical errors (2 subjects). All experiments were approved by the local ethics committee, and all the subjects provided written informed consent in accordance with the Declaration of Helsinki.

**TABLE 1:** An overview of test person groups and assessment.

	YOUNG GROUP	OLD GROUP
Participants	22	24
Sex	13 female, 9 male	13 female, 11 male
Age	18-33 years (mean 24.59 ± 3.96)	62-86 years (mean 69.42 ± 5.85)
BDI-II	median 1.5 points (IQR = 6, range 0-11)	median 4.0 points (IQR = 7, range 0-13)
MoCA	–	mean score 25.75 ± 2.07 (range 21-29 points)
Mean money gain in €	52.69 ± 1.18 (range, 50.09 – 55.25)	51.45 ± 1.57 (48.66 – 54.75)

### Stimuli and Procedure

In this study, we utilized the monetary incentive delay task (MID), which is the most frequently used paradigm for examining reward system function (Knutson et al., 2000). In this paradigm, scalable monetary cues indicate possible rewards that can be earned by a fast response to a target.

**FIGURE 1:**
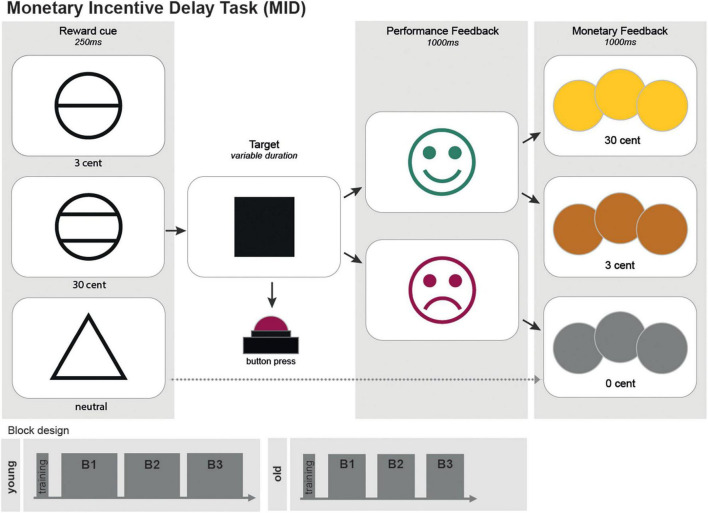
A scheme of the monetary incentive delay task (MID). The MID consisted of 450 trials (300 trials for the older group) separated into three blocks. Each trial randomly started with one of the three following reward incentive cues: a circle with one line indicated a possible gain of 3 cents; a circle with two lines, indicating a possible gain of 30 cents; and a triangle, indicating a possible gain of 0 cent. The cues were shown for 250 ms each. The trials cued with a triangle (0 cent-gain) served as the neutral control condition. Between the cue and the target, a fixation cross with randomized duration (750-1,250 ms) was presented. Target duration was adapted to individual reaction times, resulting in a predestined hit rate of 75%. Performance feedback was presented by a green laughing smiley if the time limit was not exceeded. A red sad smiley appeared otherwise. Afterward, pictures of coins visualized monetary rewards. Both feedback screens had duration of 1,000 ms each.

The here employed version of the MID task (Figure 1) consisted of 450 trials (300 trials for the older group), subdivided into three blocks of 150 (100) trials with short breaks in between (Figure 1). Each trial randomly started with one of the three following reward incentive cues:

A circle with one line indicated a possible gain of 3 cents.
A circle with two lines, indicating a possible gain of 30 cents.
A triangle, indicating a possible gain of 0 cent. Cue were shown for 250 ms each. The trials cued with a triangle (0 cent - gain) served as the neutral control condition.

The participants were told to hit the answer button with their right index fingers as fast as possible as soon as a white square (the target) appeared on the screen afterward. Between the cue and the target, a fixation cross with randomized duration (750-1,250 ms) was presented. Target duration adapted to individual reaction times, resulting in a predestined hit rate of 75%. Performance feedback was presented by a green laughing smiley if the time limit was not exceeded. A red sad smiley appeared otherwise. Afterward, pictures of coins visualized monetary rewards. Both feedback screens had duration of 1,000 ms each. For investigating neural mechanisms of reward prediction error, in 50 (33 for the older group) trials, unexpectedly, no monetary rewards were distributed. Gain cues preceded these trials, and the participants hit in time (positive feedback), but, on the last screen, a gray coin was displayed (not analyzed in this study). The participants received a start budget of 20€ (30€) and could earn about 30€ (20€). They were told in the beginning that the money they earned (Table 1) would be paid as an expense allowance by non-cash payment afterward. Test persons performed a short training session in advance.

Measurements took place in a magnetically shielded chamber of the Biomagnetic Centre, Compartment of Neurology, in the University Hospital of Friedrich-Schiller-University Jena. EEG was conducted using MEG-compatible 60-channel EEG caps (Waveguard, ANT). Stimuli were presented on a screen in the chamber using Presentation (Neurobehavioral Systems, Inc., Berkeley, CA., United States, Version 16.3). The participants responded with a keyboard (a LUMItouch photon control optical response pad). The participants had sufficient or corrected-to-normal vision.

### Preprocessing of EEG Data

**FIGURE 2:**
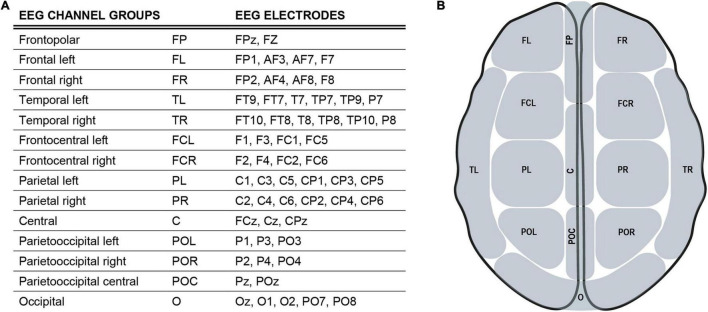
EEG channel groups **(A),** an overview of EEG channels and their assignment to regional channel groups. **(B)** Simplified scheme of EEG channel groups defined in 2 **(A)**.

EEG data were acquired using a sampling rate of 1 kHz. A bandpass filter for.1–1,000 Hz was applied. Measurement data were preprocessed with the MatLab fieldtrip toolbox (Oostenveld et al., 2011). Measurement files were segmented into trials lasting from −.5 to 1. s around the trigger onset. These trials were entirely employed for subsequent analyses. The data were downsampled to 500 Hz and then submitted to a visual artifact correction method, reject visual. Bad EEG channels and trials with artifacts were removed. An independent component analysis (ICA) was performed to correct for eyeblink and heartbeat artifacts, as well as electric noise. Finally, a bandpass filter for.1–100 Hz was applied. To provide analysis with respect to functional segregation of cortical areas, EEG channels were anatomically divided into 14 groups corresponding to their underlying brain regions (Figures 2A,B).

Connectivity analyses (Friston, 2011; Sakkalis, 2011) were applied by estimating coherence with the fieldtrip toolbox (Oostenveld et al., 2011), employing a non-parametric approach event-related for frequencies of 1. to 40 Hz (in 1-Hz steps) for all combinations of channel groups (Pereda et al., 2005). First, data were Fast Fourier transformed (Cooley and Tukey, 1965) using DPSS (discrete prolate spheroidal sequences) as a tapering function. Then, the cross-spectral density (Formula 1) was computed from frequency domain data. Finally, using the cross-spectral density matrix, the coherence coefficient C_*XY*_(f) for signals X and Y representing pairwise channel-group combinations was calculated (Formula 2)^1^.

**FORMULA 1:** Calculation of cross-spectral density of Fourier-transformed data X (f) and Y (f) (Kida et al., 2015).

*G*_*xy*_(*f*) = *X*(*f*)*Y^T^*(*f*)

**FORMULA 2:** Coherence coefficient; x (t), y (t) - time series; Gxy (f) - cross-spectral density of x and y; Gxx (f), Gyy (f) - autospectral densities of x and y (Kida et al., 2015).

$C_{xy}\left(f\right)=\frac{{\left|G_{xy}\left(f\right)\right|}^{2}}{G_{xx}\left(f\right)G_{xy}\left(f\right)}$

As coherence analysis here was conducted on the channel group level, spatial resolution was reduced in advance to serve asgreater reliability of results. Averaging of signals across these regional channel groups facilitates dissociation of adjacent regions and reduces short-interelectrode-distance volume conduction effects. Consequently, genuine coherence results can be expected for inter-regional coherence, especially for non-adjacent channel groups (Srinivasan et al., 2007; Fries, 2015).

### Statistical Analysis

Coherence results were analyzed using R (Version 4.1.1/Kick Things) (R Core Team, 2020) and RStudio (Version 2021.09.0 Build 351) (RStudio Team, 2020). Coherence coefficients of each channel combination were averaged across frequency bands (alpha, 8–12 Hz; beta, 13–30 Hz; delta, 0.5–3 Hz; theta, 4–7 Hz) (Engel and Fries, 2010). We were interested in group differences in the connectedness between prespecified channel groups (channel groups = networks) (Figure 2). To access the between network connectivity, the connectivity between each combination of channels of the two networks was estimated and averaged for each participant. Afterward, coherence values were transformed into a z-score by Fisher’s z-transformation. This analysis was performed separately for each experimental condition, resulting in one averaged value per network-condition subject. Within-group comparisons of the between network connectivity were performed by a paired *t*-test between the experimental conditions. To account for between-group differences, Welch’s two sample *t*-test was used by entering the connectivity difference between experimental conditions for each subject. All results were corrected for multiple comparison by using the false discovery rate (FDR).

Statistical analyses of questionnaire data were conducted using SPSS software (Version 27, IBM). If the normal distribution was not given, nonparametric tests were employed. Reaction times were resolved by excluding misses and values below 150 ms as well as all reaction times above 414 ms (75. quartile plus 3*IQR) as outliers (Baayen and Milin, 2010; Marković et al., 2019).

To analyze reaction times, generalized estimating equations (GEE) assuming a normal-distributed response (Liang and Zeger, 1986; Højsgaard et al., 2005) were calculated using R (Version 4.1.1/Kick Things) (R Core Team, 2020) and RStudio (Version 2021.09.0 Build 351) (RStudio Team, 2020). The goal of GEE is to draw inferences from the population by accounting for the within-subject correlation of longitudinal data. Ignoring these correlations would lead to regression estimates, being more widely scattered around the true population means. It is an extension of the generalized linear model (GLM) to correlated data, enabling the calculation of valid standard errors of the parameter estimates.

Based on the three parameters cue (reward incentive; control, 3ct, 30ct), block (B1, B2, B3), and group (young and old) and their interaction terms, different parameter combinations were tested as well as the two different most reliable correlation structures (AR1, exchangeable) to find the most suitable GEE model for the data. After testing different models (Supplementary Table 2) Model M3 has the lowest Model selection criteria values and is used for further analyses (Supplementary Tables 3, 4).

## Results

The participants performed the MID task while undergoing EEG measurement and reaction time recording. Reward sensitivity is considered as faster reaction times with increasing reward.

### Behavioral Data

**FIGURE 3:**
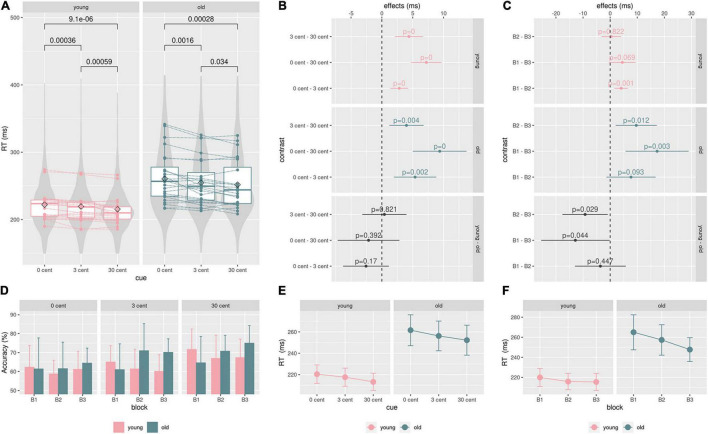
Behavioral data: **(A)**, Boxplot: single mean reaction time data, lines/points correspond to different participants. P-values from the paired Mann-Whitney U-Test show significant reward-related reaction time speeding within both groups. Violin plots are based on single reaction time data. **(B)** and **(E)**, the GEE model results for effects of the groups (young, old) and the cue (0 ct, 3 ct, 30 ct) on reaction times. Reaction time differences between cues did not significantly differ between groups. **(D)**, a hit rate (accuracy) in percent for each block and condition. Older adults show higher hit rates than younger adults during the second and third blocks across all conditions. **(C)** and **(F)**, analyzing the effect of the blocks on reaction times showed that the older adults significantly improved performance from Blocks B1 to B3 and B2 to B3. These improvements are significantly higher in comparison to the younger adults. The younger adults showed only a significant improvement between Blocks B1 and B2, not significantly differing from the older group; color code: green = old, pink = young, gray = contrast.

Analyzing reaction times, mean reaction times decreased with increasing monetary incentives in the younger and in the older group (control, 3 ct, 30 ct, Figure 3A, full data: Supplementary Table 1). Figure 3A gives an overview of the data, showing significant reward-related reaction time reduction within both groups (Mann-Whitney-U-Test). Notably, there was a higher standard deviation in RTs in the older group, being a well-known finding (Woods et al., 2015).

The GEE model revealed significant reward-related reaction time reduction effects within both groups (Figure 3B model contrasts, Figure 3E estimated marginal means, see Supplementary Table 3 for exact values). But there were no significant differences in the cue contrast between both groups (Figure 3B).

According to the model, the participants of the older group generally reacted 46.66 ms (SE = 10.3, CI: 26.5-66.8, *p* < .001) slower than the participants of the younger group. Analyzing reaction times considering the blocks revealed that the older adults significantly improved reaction times from Blocks B1 to B3 and from B2 to B3. These improvements were significantly higher in comparison to younger adults (Figure 3C model contrasts, Figure 3F estimated marginal means, see Supplementary Table 4 for exact values). Younger adults only exhibited a significant improvement between Blocks B1 and B2, not significantly differing from the older group. Older adults show higher hit rates than younger adults during the second and the third block across all conditions (Figure 3D). In an additional regression analysis, no significant effect of the factor age on reaction time differences between conditions could be found (Supplementary Figure 1).

### Effects of Rewards on Frontal Intra- and Internet Work Connectivity

**FIGURE 4:**
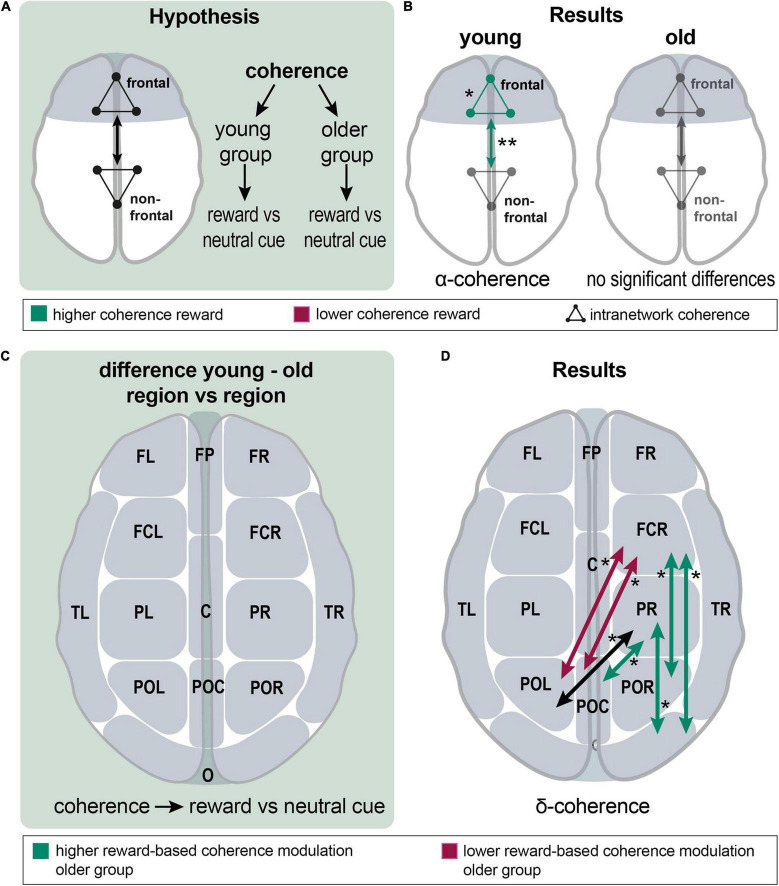
Analysis of reward effects on frontal intra- and internetwork connectivity and of group differences in reward effect on interregional connectivity; **(A),** a schematic overview over the analyzing procedure: Within-group coherence was analyzed for the reward vs. neutral cue contrast between frontal and non-frontal EEG channel groups. **(B),** Results show a higher frontal network and higher frontal-nonfrontal alpha-band coherence during the reward cue. There were no significant differences in the older group. **(C),** A schematic overview of the analyzing procedure: Between group comparison of interregional coherence for the reward vs. neutral cue contrast. **(D),** Results: significant differences appeared in the delta band; reward-based modulation was higher in the older group for frontocentral right and parietal right with parietooccipital and occipital channel groups. In detail, this applied to FCR with POR and O; and PR with POC and O (green arrows). Modulation was higher in the younger group for FCR with POL and POC (red arrows). Coherence was significantly different between PR and POL; both groups modulated it in the same absolute value, but in different directions (a black arrow). Asterisks: one asterisk: *p* ≤ 0.05; two asterisks: *p* ≤ 0.01.

For investigating reward effects, a within-group comparison of the neutral vs. high-reward cue (0 ct vs. 30 ct) and neutral vs. high-reward feedback (0 ct vs. 30 ct) (Figures 4A,B) was conducted. Important network centers of the reward system are represented in the prefrontal cortex (Kringelbach, 2005; Tobler et al., 2009; Doñamayor et al., 2012; Li et al., 2016). Thus, we expected higher reward-related frontal connectivity. All frontal channel groups (FR, FL, FP, FCL, FCR, see Figure 2) and all remaining channel groups each were summarized into a new EEG channel group. Connectivity among the five frontal channel groups and between the newlydefined frontal and non-frontal channel groups was assessed (Figure 4A).

First, the reward cue effect on frontal connectivity was analyzed. In the young group, coherence within the frontal group (mean = 0.434 vs. 0.421; *p* = 0.012; df = 21) and frontal-non-frontal coherence was significantly higher in the alpha band for the reward cue (mean = 0.405 vs. 0.391; *p* = 0.002; df = 21). In the beta band, a non-significant tendency for higher coherence during the reward cue appeared within the frontal group (*p* = 0.070) and between the frontal and non-frontal groups (*p* = 0.056). In the older group, no significant differences existed for frontal-nonfrontal coherence and coherence among frontal channel groups. There was a non-significant tendency for lower coherence during the reward cue among the nonfrontal channel groups in the delta band (*p* = 0.061) (Figure 4B). In a finer-grained regional analysis, significant changes only appeared in the alpha and delta bands of the young group (Supplementary Figure 2).

### Group Differences of Reward Effects on Interregional Connectivity

Interregional connectivity modulation was compared between groups to test for group differences of reward effects (Figure 4C).

Significant differences could exclusively be found in the delta band, where a mixed picture concerning differences in coherence between neutral and reward cues appeared (Figure 4D). Reward-based modulation was higher in the older group for frontocentral right and parietal right with parietooccipital and occipital channel groups. In detail, this applied for FCR with POR and O; and PR with POC and O. Modulation was higher in the younger group for FCR with POL and POC. Noteworthy, younger and older adults exhibited connectivity modulations in different directions, such as coherence increased for reward compared to neutral cues in the younger group, whereas it decreased in the older group. In this way, coherence was significantly different between PR and POL; both groups modulated it in the same absolute value, but in different directions.

### Application of Cognitive Aging Models on Reward Processing in Healthy Aging

#### HAROLD

**FIGURE 5:**
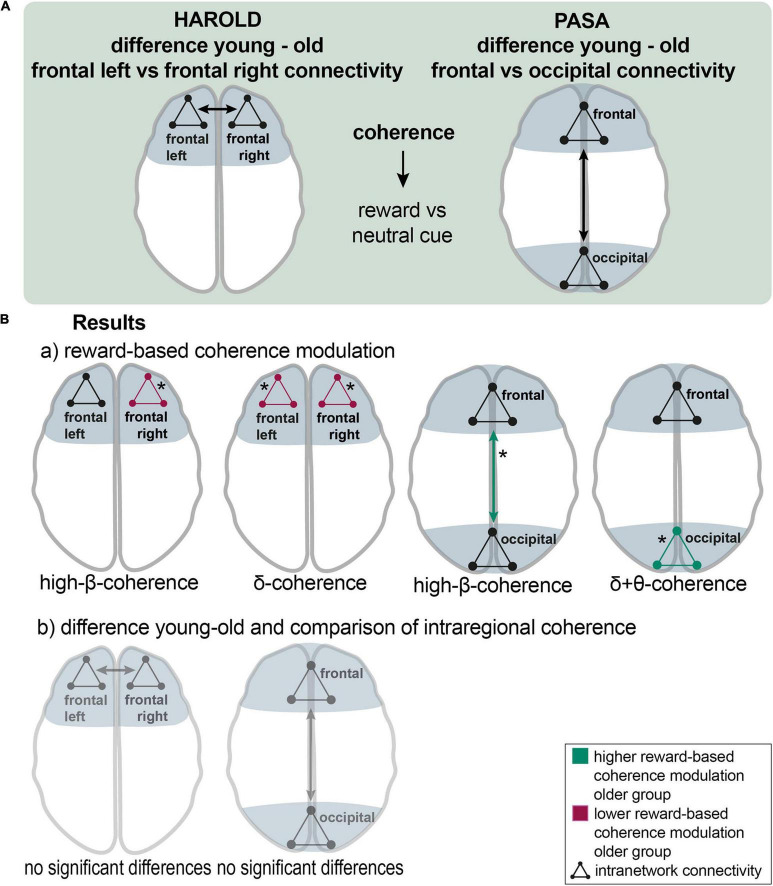
The HAROLD and PASA effect during reward processing: **(A),** a schematic overview of the analyzing procedure: Testing HAROLD (left side), group differences in coherence between frontal left and frontal right channel groups were assessed for the reward vs. neutral cue contrast. Testing PASA (right side), group differences in coherence between frontal and occipital channel groups were assessed for the reward vs. neutral cue contrast. **(B),** Results: a) HAROLD in the delta band, the older group exhibited lower reward-based coherence modulation within the left frontal and the right frontal channel groups. In the high-beta band, the older group showed lower differences in coherence within the right frontal channel group than the younger group (red arrows). PASA - the difference between conditions was greater in the older compared to the younger group for coherence among occipital channel groups in the delta band and in the theta band. In the high-beta band, the older group exhibited a significantly higher reward-based modulation in coherence between frontal and occipital groups (green arrows). b) HAROLD - to investigate hemispheric asymmetry, the difference in reward-based coherence modulation of the frontal left and the frontal right channels was compared between the older and the younger groups, revealing no significant results. PASA - comparing the difference in reward-based modulation of frontal and occipital coherence between groups, no significant results appeared. Asterisks: one asterisk: *p* ≤ 0.05.

In case of the HAROLD model (hemispheric asymmetry reduction in older adults) (Cabeza, 2002), frontal connectivity within and between the frontal areas of both hemispheres was assessed and compared between groups for the cue events. Therefore, two new channel groups were defined, including all frontal cortical channel groups of the left or right hemispheres (left: FL, FCL; right: FR, FCR) (Figures 2, 5A).

In the delta band, the older group exhibited lower reward-based coherence modulation within the left frontal [mean = −0.041 vs. 0.019; *p* (uncorr) = 0.046; df = 30.1] and the right frontal channel group [mean = −0.05 vs. 0.009; *p* (uncorr) = 0.017; df = 41.8]. The younger group exhibited higher reward-based coherence modulation in frontal left with all other channel groups in the alpha band (mean = 0.013 vs −0.07; *p* = 0.038; df = 32). In the high-beta band, the older group similarly showed lower differences in coherence within the right frontal channel group [mean = 0.014 vs. 0.003; *p* (uncorr) = 0.035; df = 40.5] than the younger group. In the high-beta band, there was a non-significant result for higher modulation in the younger group between frontal right and other channel groups (*p* = 0.062) (Figure 5B,a). To investigate hemispheric asymmetry, the difference in reward-based coherence modulation of the frontal left and the frontal right channels was compared between the older and the younger groups, revealing no significant results (Figure 5B,b).

#### PASA

For testing the PASA (posterior-anterior shift in aging) model (Davis et al., 2008), an analysis of connectivity between and among frontal and occipital channel groups was conducted and compared between the older and the younger groups for the cue events. Therefore, frontal channel groups (FP, FR, FL, FCR, FCL) and parietooccipital and occipital channel groups (POC, POL, POR, and O) were summarized into two new groups (Figures 2, 5A).

The difference between conditions was greater in the older compared to the younger group for coherence among occipital channel groups in the delta band [mean = 0.001 vs. −0.01; *p* (uncorr) = 0.044; df = 33] and in the theta band [mean = 0.001 vs. −0.006; *p* (uncorr) = 0.043; df = 29.3]. In both cases, coherence was modulated reversely in older adults so that it decreased during reward cues. In the beta band, the older adults showed higher coherence modulation between occipital and all other channel groups (mean = 0.001 vs. −0.004; *p* = 0.045; df = 37.4). In the high-beta band, the older group exhibited a significantly higher reward-based modulation in coherence between frontal and occipital groups [mean = 0.003 vs. −0.006; *p* (uncorr) = 0.039; df = 34.2] and a marginally non-significant result for differences in coherence among occipital channel groups [*p* (uncorr) = 0.059]. In the beta band, two non-significant results pointed to a greater difference in coherence between frontal and occipital groups [*p* (uncorr) = 0.061] and in coherence among occipital channel groups [*p* (uncorr) = 0.052] in the older group. A non-significant tendency appeared in the alpha band, indicating lower differences between conditions in coherence between frontal and occipital groups [*p* (uncorr) = 0.066] and between frontal and remaining channel groups (*p* = 0.060) coherence in the older group (Figure 5B,a). Comparing the difference in reward-based modulation of frontal and occipital coherence between groups, no significant results appeared (Figure 5B,b).

#### Frontoparietal Control Network

**FIGURE 6:**
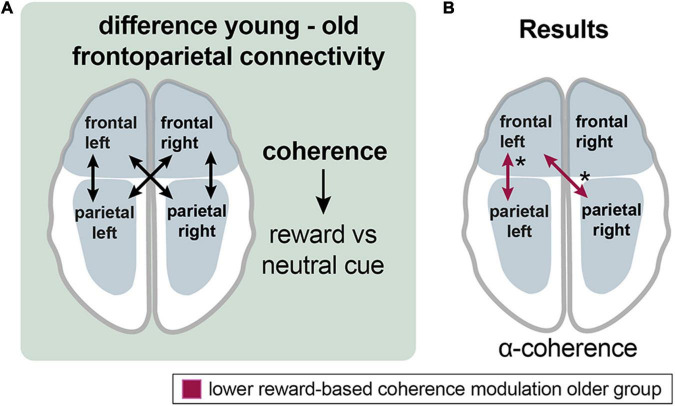
Aging effects on the frontoparietal control network during reward processing**. (A)** Analyzing the procedure scheme: group differences in coherence between frontal and parietal channel groups were assessed for the reward vs. neutral cue contrast. **(B)** Results: Older adults exhibited lower reward-related frontoparietal left and frontal left-parietal right coherence modulation in the alpha band (red arrows). Asterisks: one asterisk: *p* ≤ 0.05.

To investigate connectivity between frontal and parietal areas for the cue events, four new channel groups were defined: a frontal right (FR, FCR), a frontal left (FL, FCL), a parietal left (PL, POL), and a parietal right channel group (PR, POR) (Figures 2, 6A).

In the alpha band, the older group exhibited lower left frontoparietal [mean = 0.013 vs. −0.005; *p* (uncorr) = 0.043; df = 35.3] and left frontal with right parietal [mean = 0.015 vs. −0.01; *p* (uncorr) = 0.032; df = 34.1] reward-based coherence modulation compared to the younger group (Figure 6B).

#### Questionnaire

Analysis of questionnaire data amounted to the following results (Table 1). To test for depressive symptoms, the BDI-II score was chosen. Across subjects, the median BDI-II score (Beck et al., 1996) was 1.5 (IQR = 6; range, 0-11) in the younger and 4 (IQR = 7; range, 0-13) in the older group. The Mann-Whitney U-Test for independent samples (*p* = 0.349; U = 222) showed no significant difference between groups. Concerning the MoCA test (Nasreddine et al., 2005), the mean score was 25.75 (SD = 2.07; range, 21-29 points), slightly below the original cut-off score of 26 points (results < 26 indicate cognitive impairment) (Nasreddine et al., 2005). Test results showed a normal distribution (Shapiro-Wilk Test, *p* = .349). However, no participant was excluded based on the results of the MoCA test. Moreover, we carefully checked for correct understanding and execution.

For hedonia assessment, the items stress, gambling behavior, religiosity, pack-years of smoking (py), and alcohol consumption (Saunders et al., 1993) of the anamnesis questionnaire were evaluated. For religiosity and stress, the participants had to answer on a scale from 1 to 10 (1 – not stressed/not religious, 10 – high stress/very religious). Both groups did not differ in concerns of religiosity (*p* = 0.319; U = 211.5) and alcohol consumption (*p* = 0.781; U = 248). Significant differences for the items stress (*p* ≤ 0.000; U = 57.5) and py (*p* = 0.045; U = 182.5) were found, revealing that the younger participants suffered significantly more from stress and smoked significantly less than the older participants.

## Discussion

### Behaviorally Robust Reward Sensitivity in Older Age

In line with our hypothesis, we did not find a significant effect of age on reward-dependent modulation of reaction times (RTs). Younger and older adults significantly decreased their reaction times with increasing reward (Figure 3 and Supplementary Table 1). The decrease in reaction times with an increasing monetary incentive reflects an intact reward sensitivity in aging. This overall stability might be an explanation for well-known psychological changes in aging. Older adults tend to aim at preserving resources, preventing negative outcomes and maintaining their emotional well-being. This refers to socioemotional selectivity theory (SST), which emphasizes older adults’ most prioritized goals of sustaining positive affect due to perceiving their further lifetime as limited (Carstensen et al., 1999). Thus, older people pay more attention to positive compared to negative stimuli. This so-called “positivity effect” explains an empirical phenomenon, stating that information processing exhibits a positive bias in aging (Mather and Carstensen, 2005; Reed et al., 2014).

Our results of a preserved sensitivity in older adults are in line with most aging aging studies employing the monetary incentive delay task (MID) (Samanez-Larkin et al., 2007; Rademacher et al., 2014; Vink et al., 2015). However, some studies reported a preserved but reduced or total lack of reward sensitivity in older adults (Spaniol et al., 2015; Dhingra et al., 2020). An explanation for these differences could be the differing number of trials and investigated subjects (Samanez-Larkin et al., 2014; Yee et al., 2019). Studies reporting reduced reward sensitivity generally conduct fewer trials and, often, foregoing training sessions prior to the actual experiment (Spaniol et al., 2015; Dhingra et al., 2020). These differences indicate that older adults might need a longer time for getting used to a task so that differences in task performance stand out when the experimental duration is shorter. Although this training effect might account for reports of significant differences in age-related reward sensitivity, it is, nevertheless, possible that there is a small effect of age on reward sensitivity that might require a high number of participants and long experiment duration to become statistically significant. Longer experiment durations, however, increase the risk that any measured effects are no longer primarily due to the principal performance of the reward system but to faster age-related exhaustibility of the attentional system. Regardless of these methodological difficulties, a possible effect of age on the functionality of the reward system must be small compared to other cognitive and behavioral domains (particularly in comparison to the age-related decrease in RTs). Thus, it remains an open question how the aging brain manages to preserve reward system functionality despite the cellular and molecular changes affecting its core parts. Here, we conducted further analyses to address the question of what compensatory mechanisms are used to maintain reward system function in the elderly.

Here, we used EEG connectivity measures to clarify this question. To our best knowledge, no previous study investigated the aging reward system with EEG connectivity measures (Meyer et al., 2021). Connectivity analyses further improve the understanding of reward network integrity and communication of cortical brain areas during reward processing (Sakkalis, 2011) and were here performed with particular focus on the prefrontal cortex as a center for important network hubs of the reward system (Kringelbach, 2005; Tobler et al., 2009; Doñamayor et al., 2012; Li et al., 2016).

### Reward Prediction in Young and Older Adults

Younger and older adults showed different reward-related connectivity patterns (Figure 4B and Supplementary Figure 2). During reward prediction, the younger adults showed significantly higher coherence in the alpha band and lower coherence in the delta band (Figure 4B and Supplementary Figure 2), while, in the older group, there was no significant reward-based modulation of coherence. The distribution of alpha-band changes is in line with the current literature, suggesting altered activity within the frontoparietal control network (Sadaghiani and Kleinschmidt, 2016; Lepage and Vijayan, 2017) and the ventral attention network (VAN) (Solís-Vivanco et al., 2021). Both have been found to be involved in the reorientation of attention and the maintenance of selective attention (Corbetta and Shulman, 2002; Mengotti et al., 2020). Thus, our findings imply that younger adults show enhanced recruitment of FPCN and VAN as a sign of cognitive control and increased attention in a rewarding context (Corbetta and Shulman, 2002; Persichetti et al., 2015). With respect to the decreased delta band coherence, it has been suggested that the cues elicited a “cognitive reward” and, therefore, caused a reward feedback-like delta band response (Bromberg-Martin and Hikosaka, 2009; Wang et al., 2016). Taken together, changes in alpha-band coherence might suggest increases in cognitive control and attention in a rewarding context in young adults, while delta band coherence during non-reward cues in younger adults may resemble a negative reward due to the absence of a possible monetary gain. The absence of such a modulation in older adults might reflect their reduced ability to flexibly recruit cognitive control mechanisms in response to varying incentives (Huizeling et al., 2021).

Significant changes in the delta band were also found by comparing the reward-related connectivity patterns between older and younger adults (Figure 4D).

### Analysis of Group Differences in Reward Effects on Interregional Connectivity

Comparing the reward-related connectivity patterns between older and younger adults, significant differences only emerged during the cue events in the delta band. The younger adults exhibited higher reward-based modulation of coherence in the frontal right with parietooccipital left channel groups (Figure 4D). Older adults exhibited higher frontoparietal right and parietooccipital coherence modulation. Noteworthy, younger and older adults exhibited connectivity modulations in different directions, such as coherence increased for reward compared to neutral cues in the younger group, whereas it decreased in the older group (Figure 4D).

According to the function of delta oscillations, they support the idea of older adults’ altered pathways of detecting and processing salient stimuli (Knyazev, 2007; Knyazev, 2012; Daitch et al., 2013; Güntekin and Başar, 2016). Especially, older adults’ higher reward-related coherence modulation in the delta band resembles the ventral attention network (VAN) (Corbetta and Shulman, 2002). It consists of the temporoparietal junction and middle and inferior frontal gyrus, exhibiting a right-lateralized activation. The VAN also underlies connectivity alterations in aging, although studies disagree on their direction and magnitude (Li et al., 2015; Kurth et al., 2016; Deslauriers et al., 2017; ElShafei et al., 2020). One study reported increased connectivity within the VAN in older adults, which is in line with our findings (Deslauriers et al., 2017). These age differences in the reward-related network connectivity likely reflect compensatory mechanisms employed by the aging brain to maintain behavioral reward system function.

### Application of Cognitive Aging Models on Reward Processing

Age-related cognitive network alterations are well-observed by fMRI in older adults and comprise hypo- and hyperactivations in specific patterns that have been summarized in distinct models. Their functional effect has been interpreted in the sense of dedifferentiation and compensation (Sala-Llonch et al., 2015). Two of the best established compensatory models are the HAROLD (Hemispheric Asymmetry Reduction in Older Adults) (Cabeza, 2002) and the PASA (posterior-anterior shift in aging) model (Davis et al., 2008). Furthermore, frontoparietal control network (FPCN) hyperactivation has been observed in older adults during cognitively demanding tasks (Reuter-Lorenz and Park, 2014; Li et al., 2015). The FPCN exerts a paramount role in organizing cognitive control and goal-directed behavior (Spreng et al., 2010; Parro et al., 2018). Due to the close link between reward processing and cognition, we propose the involvement of HAROLD, PASA, and FPCN compensatory mechanisms in preserved reward system function in healthy aging. These compensatory models have been tested successfully with EEG connectivity analyses elsewhere (Rosjat et al., 2018; Rosjat et al., 2021).

Now, these models of cognitive aging were tested regarding their applicability to the aging reward system (Figure 5A) (Cabeza, 2002; Reuter-Lorenz and Mikels, 2006; Davis et al., 2008; Reuter-Lorenz and Park, 2014; Festini et al., 2018).

First, the HAROLD theory (Hemispheric Asymmetry Reduction in Older Adults) was applied (Figure 5B). It suggests the bilateral recruitment of prefrontal brain areas in older adults compared to a predominantly lateralized activity in younger adults during the same cognitive task as a compensatory mechanism (Cabeza, 2002; Cabeza and Dennis, 2012). The additional activation is related to performance improvements in older adults (Cabeza and Dennis, 2012). In the delta and high-beta bands, the older group showed lower reward-related modulation of bilateral intrahemispheric frontal coherence than the younger group during the cue events. However, the direct test for hemispheric asymmetry yielded no significant results (Figure 5B,b). Hemispheric asymmetry reduction in older adults has been reported during cognitive tasks across studies (Hogan et al., 2012; Learmonth et al., 2017; Kenney et al., 2019; Rosjat et al., 2021). The here presented results do not show an apparent HAROLD effect. A possible explanation might be that older adults rely less on the frontal cortex for reward processing, as indicated by the results reported above. Nevertheless, a HAROLD effect could be observable in other cortical areas that were not analyzed here (Kenney et al., 2019).

Another consistently observed pattern describes a prefrontal over- related to an occipital underrecruitment in older compared to younger adults, resulting in the PASA model (Davis et al., 2008; Figure 5A). The additional prefrontal activation in older adults is argued to be a compensatory mechanism for age-related occipitotemporal deficits in the processing of sensory stimuli (Grady et al., 1994). Evidence indicates that occipital activity is negatively correlated with frontal activity, and the latter correlates positively with cognitive performance, providing a mechanism for compensation (Davis et al., 2008; Cabeza and Dennis, 2012). During the cue events, the older adults exhibited higher reward-based modulation of occipital coherence in the delta and theta bands and of fronto-occipital coherence in the high-beta band. Contrary to the PASA hypothesis, the results show higher reward-related occipital coherence modulation in the older group during reward anticipation. This is in line with the above-reported results, indicating that older adults rely less on frontal cortical areas during reward processing. Explicit comparison of frontal relative to occipital connectivity between groups amounted to no significant differences (Figure 5B,b). Thus, the results show no clear PASA effect. Nevertheless, the higher fronto-occipital coherence modulation in the older group could comply with a PASA effect (Rosjat et al., 2021). The PASA phenomenon has been described during cognitive tasks across studies (Huizeling et al., 2021; Rosjat et al., 2021; Seider et al., 2021). According to the PASA hypothesis, declined occipital activation is due to older adults’ sensory processing deficits.

To sum up, we found no evidence that the HAROLD or PASA model explains significant portions of age-related compensatory mechanisms in the reward system, indicating that they might only apply to specific cognitive tasks, and not to the aging reward system.

### Lower Reward-Related Frontoparietal Control Network Modulation in Aging

Studies to date especially focused on aging effects on the reward system itself, whereby neglecting incentive-based modulation of the previously described large-scale brain networks (Spaniol et al., 2015). Reward-dependent performance improvement is mediated by the interaction of the reward system with large-scale cognition-related networks, such as the FPCN or the VAN, by implementing cognitive control (Spaniol et al., 2015; Parro et al., 2018). Incentive-based overrecruitment of the default-mode network and cognitive control regions in older compared to younger adults together with similar reward-network activation in both groups has been reported (Spaniol et al., 2015).

An underlying mechanism of performance enhancement under reward conditions is the functional interaction of the reward network with cognition-related large-scale networks like the frontoparietal control network (FPCN) (Vincent et al., 2008), default network (DN), and ventral and dorsal attention network (VAN, DAN). These are engaged, respectively, and disengaged and interconnected during the performance of cognitive tasks, according to task demands (Spaniol et al., 2015; Parro et al., 2018). Importantly, the older adults achieved equal performance as the younger adults when activation levels in the frontoparietal control network were higher (Li et al., 2015). Therefore, we hypothesized frontoparietal hyperactivation in the older adults as a compensatory mechanism for functional deficits of other brain areas (Reuter-Lorenz and Park, 2014), implementing pronouncement of cognitive control processes (Parro et al., 2018). Testing reward-related frontoparietal coherence modulation (Figure 6A), the older group showed lower reward-based modulation of left frontoparietal and left frontal with right parietal alpha-band coherence during cue events (Figure 6B). Thus, our hypothesis could not be confirmed as the opposite effect was observed.

Conclusively, we found age-related compensatory mechanisms that did not fit to predescribed age-related compensatory mechanisms of other cognitive domains. Thus, we propose that different neural mechanisms underlie the preserved behavioral function of the aging reward system. One reason for this could be the large evolutionary gap in development between the reward system and higher cognitive functions. Evolutionary preserved reward-related delta oscillations support this idea (Knyazev, 2012). Further evidence shows that salience networks underlie less age-related decline compared to other cognitive networks (Zhang et al., 2014). For instance, fear as another important evolutionary skill for survival has also been found to be relatively stable in healthy aging (LaBar et al., 2004).

### Methodical Limitations

Possible limitations may have been the small number of test persons. To identify older participants with mild cognitive impairment up to dementia, the MoCA test was conducted, resulting in a mean score of 25.75 (range, 21-29) (Table 1). The original cut-off for a normal result is ≥ 26 points, which has often been criticized for being too conservative (Thomann et al., 2018); therefore, no participant was excluded based on this test.

Task performance in the MID may be compromised by a large number of trials, especially in EEG studies and the monotonous course of the MID (Bjork et al., 2010). Additionally, the ability to maintain attention might be reduced in older adults. Consequently, the number of trials was reduced in the older group. According to Spaniol et al., the MID only requires low task-performance skills (Spaniol et al., 2015). Therefore, they argue that aging effects on incentive processing are less confounded by aging effects on task performance (Spaniol et al., 2015). This idea is supported by studies reporting equal performance of younger and older adults in the MID using explicit reward cues, which do not require learning (Samanez-Larkin et al., 2007). Thus, the here used MID is a convenient paradigm for investigating aging of the reward system (Spaniol et al., 2015). As our aim was to shed light on aging effects on the reward system itself and reduce the influence of aged cognitive abilities, the MID is the most appropriate paradigm as it requires low cognitive processing while eliciting robust reward network activation (Spaniol et al., 2015). Although HAROLD and PASA have previously mainly been used to explain compensation in the field of cognitive aging, both were successfully tested in studies investigating risk-taking or emotional perception, being closely related to reward processing (Lee et al., 2008; Jacques et al., 2013). Furthermore, due to the close link between reward and cognitive networks, we assumed that comparable compensatory mechanisms might occur in the aging reward system.

Another point is the use of money as a secondary reward (Bonner and Sprinkle, 2002; Knutson and Wimmer, 2007; Kohls et al., 2009; Knutson and Heinz, 2015). Highly varying individual attitudes should not be neglected (Lutz and Widmer, 2014), including the participants’ current financial situation. In addition, older adults, in general, seem to prefer social and positive affective rewards over monetary rewards (Rademacher et al., 2014; Samanez-Larkin and Knutson, 2015; Dhingra et al., 2020).

Limitations of EEG are mainly represented by volume conduction, the extension of electric fields in tissues surrounding the brain, leading to low-pass spatial filtering of signals (Srinivasan et al., 1998; Nunez and Srinivasan, 2006; Srinivasan et al., 2007; Kayser and Tenke, 2015b; Bastos and Schoffelen, 2016). In case of coherence, the abovementioned spatial filtering caused by volume conduction is the main reason for artificial coherence results between EEG channels, which can be reduced by surface Laplacian transform (Srinivasan et al., 1998). Disadvantageously, it distorts signals from extensive sources and may compromise genuine coherence from widespread neural activities over a greater distance (Srinivasan et al., 1998; Nunez and Srinivasan, 2006; Srinivasan et al., 2007; Kayser and Tenke, 2015a,b). As this method was not applied in this study, especially significant coherence results of adjacent regions have to be interpreted cautiously. In sum, when interpreted carefully, coherence offers a valuable and frequently used method for investigating neuronal communication (Srinivasan et al., 2007; Fries, 2015; Bastos and Schoffelen, 2016).

## Conclusion

In the current study we found that (1) older adults show greater reliance on posterior cortical areas for reward processing, (2) reward-related connectivity modulation tends to be lower in older adults, and (3) older adults modulate connectivity in the opposite direction than younger adults, with usually greater connectivity during non-reward compared to reward conditions. Furthermore, our data indicate that older adults show a more right-lateralized reward-related connectivity, in contrast to younger adults who rely more on left-hemispheric connectivity. These findings provide important new insights into age-related changes in cortical connectivity in the reward system. The mechanisms identified for maintaining reward system function in old age did not fit into previously described models of cognitive aging. We infer that the reward system has unique compensatory mechanisms distinct from other cognitive functions. Nevertheless, further studies are needed to fully understand the complexity of changes in the reward system in healthy aging.

## Data Availability Statement

The raw data supporting the conclusions of this article will be made available by the authors, without undue reservation.

## Ethics Statement

The studies involving human participant were reviewed and approved by Ethics Committee of the University Hospital Jena, Germany (REST:2019-1473-BO). The participants provided their written informed consent to participate in this study.

## Author Contributions

LO, FW, JR, JM, AS, and SB contributed to the acquisition and analysis of data. LO, FW, and CK drafted a significant portion of the manuscript and figures. LO and FW conceived the initial idea and contributed equally. All authors read and improved the final manuscript. The manuscript is part of the thesis of LO.

## Conflict of Interest

The authors declare that the research was conducted in the absence of any commercial or financial relationships that could be construed as a potential conflict of interest.

## Publisher’s Note

All claims expressed in this article are solely those of the authors and do not necessarily represent those of their affiliated organizations, or those of the publisher, the editors and the reviewers. Any product that may be evaluated in this article, or claim that may be made by its manufacturer, is not guaranteed or endorsed by the publisher.

## Footnotes

1
https://www.fieldtriptoolbox.org/tutorial/connectivity/

## Supplementary Material

Supplementary Figure 1 Pearson’s partial correlation analysis, based on single mean values of the participants, for all three RT differences versus age, controlling for the non-participating reward condition in the difference (left: 3–30 cent, controling for 0 cent, middle: 0–30 cent, controlling for 3 cent, right: 0–3 cent, controlling for 30 cent), no significant effect could be found.

Supplementary Figure 2 Analysis of interregional analysis within the young group for the reward vs neutral cue. **(A)** Corrplot with FDR-corrected *p*-values for the alpha band; **(B)** Corrplot with FDR-corrected *p*-values for the delta band.

## Abbreviations

BDI-II: Beck Depression Inventory II
C: Central
DPSS: discrete prolate spheroidal sequences
EEG: Electroencephalography
EQ-5D: European quality of life 5 dimensions
FCL: Frontocentral left
FCR: Frontocentral right
FDR: False discovery rate
FL: Frontal left
FP: Frontopolar
FPCN: Frontoparietal control network
FR: Frontal right
GEE: Generalized estimating equations
HAROLD: Hemispheric asymmetry reduction in older adults
ICA: Independent component analysis
MEG: Magnetoencephalography
MID: Monetary incentive delay task
MoCA: Montreal cognitive assessment
mPFC: Medial prefrontal cortex
O: Occipital
OFC: Orbitofrontal cortex
PASA: Posterior-anterior shift in aging
PL: Parietal left
POC: Parietooccipital central
POL: Parietooccipital left
POR: Parietooccipital right
PR: Parietal right
py: pack years of smoking
RT: Reaction time
SF-36: Short form-36 health survey
SST: Socioemotional selectivity theory
TL: Temporal left
TR: Temporal right
VAN: Ventral attention network.
