# Supplementary figures and images for: Still Wanting to Win: Reward System Stability in Healthy Aging
Source: Front Aging Neurosci. 2022 May 30;14:863580. doi: 10.3389/fnagi.2022.863580 (PMC9190761; doi:10.3389/fnagi.2022.863580)

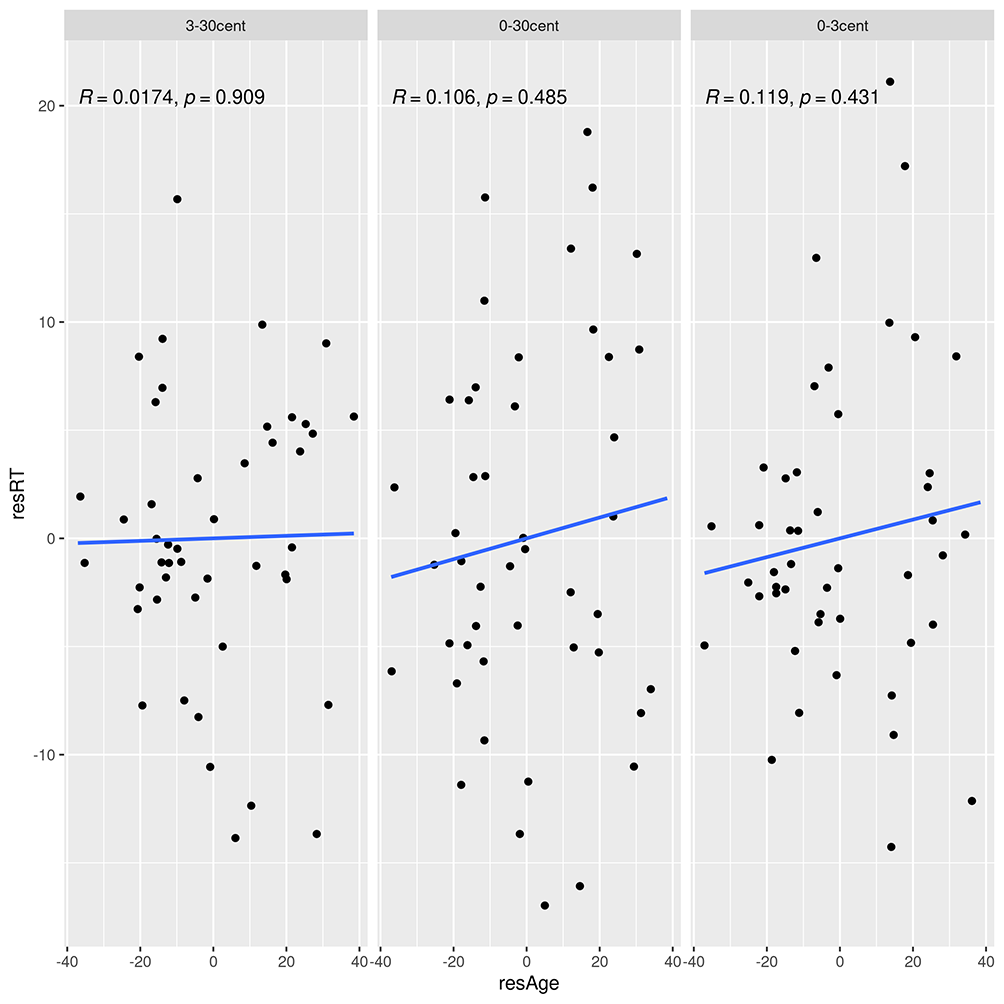

Supplement: Supplementary Figure 1 — Pearson’s partial correlation analysis, based on single mean values of the participants, for all three RT differences versus age, controlling for the non-participating reward condition in the difference (left: 3–30 cent, controling for 0 cent, middle: 0–30 cent, controlling for 3 cent, right: 0–3 cent, controlling for 30 cent), no significant effect could be found. [file Image_1.TIF]

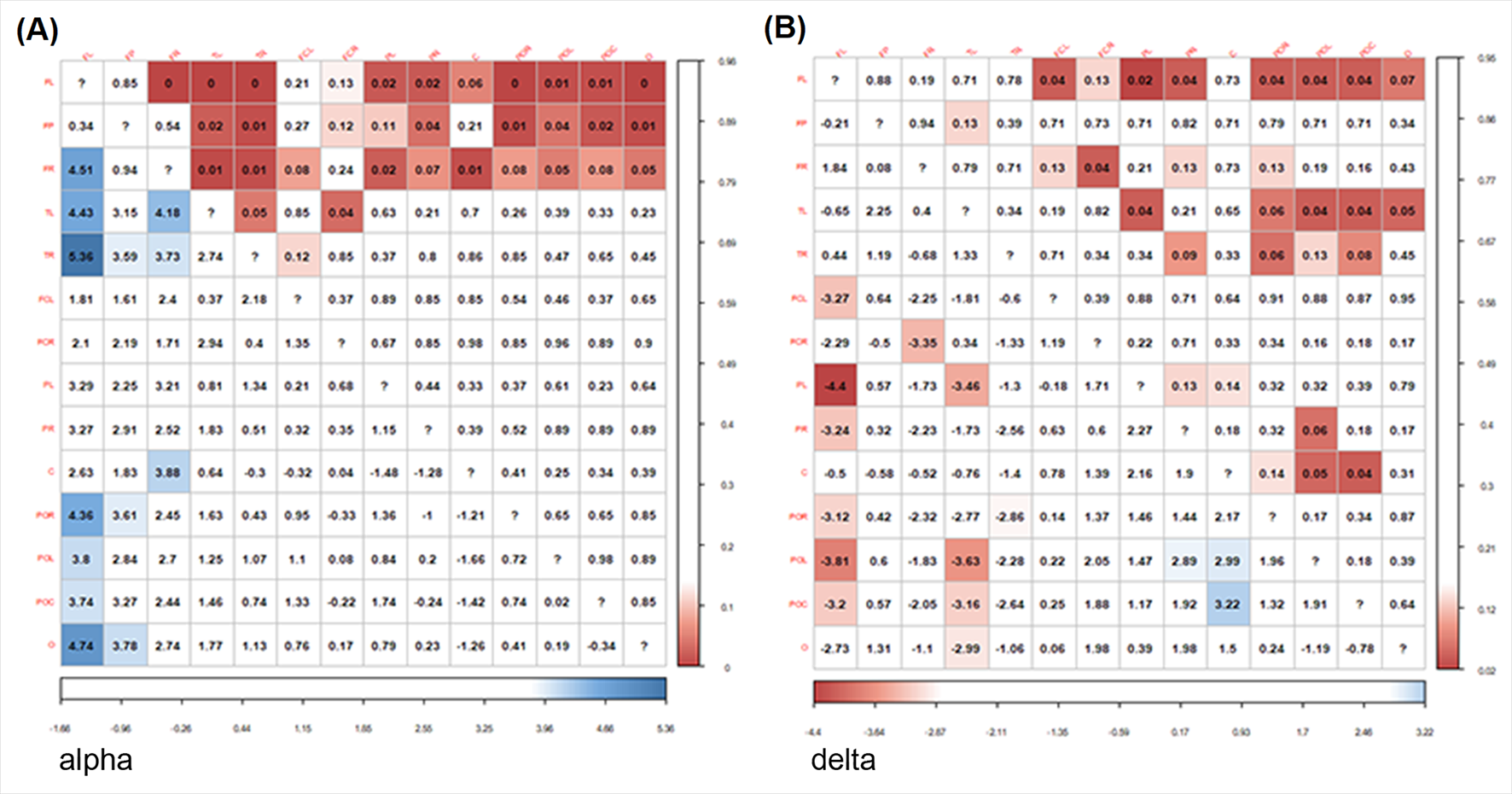

Supplement: Supplementary Figure 2 — Analysis of interregional analysis within the young group for the reward vs neutral cue. (A) Corrplot with FDR-corrected p-values for the alpha band; (B) Corrplot with FDR-corrected p-values for the delta band. [file Image_2.TIF]
