# Supplementary material for: Still Wanting to Win: Reward System Stability in Healthy Aging
Source: Front Aging Neurosci. 2022 May 30;14:863580. doi: 10.3389/fnagi.2022.863580 (PMC9190761; doi:10.3389/fnagi.2022.863580)
Supplement: Supplementary file 3 [file Table_1.docx]

|  | | Reward incentive | | |
| --- | --- | --- | --- | --- |
|  | **GROUP** | **control** | **3 cents** | **30 cents** |
| **REACTION TIME** in ms  *(mean ± STD, median IQR)* | **young** | 221.94±34.236  217.00, IQR=41 | 219.29±33.18  216.00, IQR=39 | 215.37±31.406  212.00, IQR=34 |
|  | **old** | 260.25±48.775  251.00, IQR=64 | 254.64±46.461  244.00, IQR=62 | 251.62±47.03  241.00, IQR=58 |

**Supplement Table 1**: Mean and median reaction times per group and cue
