# Supplementary material for: Still Wanting to Win: Reward System Stability in Healthy Aging
Source: Front Aging Neurosci. 2022 May 30;14:863580. doi: 10.3389/fnagi.2022.863580 (PMC9190761; doi:10.3389/fnagi.2022.863580)
Supplement: Supplementary file 4 [file Table_2.docx]

M0 M1 M2 M3 M4

--------------------------------------------------------------------------------------------------

(Intercept) 213.70 *** 223.21 *** 222.54 *** 223.28 *** 220.51 ***

(7.41) (4.51) (4.61) (4.71) (4.39)

cue3cent -1.54 -2.37 -1.50 -2.82 *** -2.79 ***

(1.18) (1.27) (1.29) (0.71) (0.71)

cue30cent -6.33 *** -7.41 *** -6.34 *** -7.21 *** -7.19 ***

(1.69) (1.81) (1.56) (1.24) (1.24)

groupold 48.78 *** 45.04 *** 46.66 *** 46.67 *** 41.08 ***

(13.46) (10.37) (10.30) (10.31) (8.54)

blockB2 4.38 -4.07 * -3.06 -4.09 ***

(8.70) (1.66) (1.57) (1.22)

blockB3 12.33 -4.27 -3.24 -4.49

(9.67) (2.68) (2.66) (2.47)

cue3cent:groupold -3.31 -0.41 -2.51 -2.57 -2.81

(2.45) (2.77) (1.86) (1.87) (1.89)

cue30cent:groupold -1.21 0.48 -2.13 -2.16 -2.43

(2.54) (2.92) (2.52) (2.53) (2.50)

cue3cent:blockB2 -2.54 -0.79 -2.01

(1.91) (2.21) (1.98)

cue30cent:blockB2 0.05 0.68 -1.04

(2.57) (2.85) (2.15)

cue3cent:blockB3 -2.15 -0.59 -2.04

(1.45) (1.49) (1.60)

cue30cent:blockB3 -0.88 -0.07 -1.64

(2.52) (2.79) (2.07)

groupold:blockB2 -14.87 -1.18 -3.60 -3.60

(12.08) (5.77) (4.74) (4.74)

groupold:blockB3 -16.62 -10.38 -12.83 * -12.83 *

(15.41) (6.82) (6.39) (6.39)

cue3cent:groupold:blockB2 1.68 -2.91

(3.47) (4.24)

cue30cent:groupold:blockB2 -3.21 -4.11

(3.74) (4.31)

cue3cent:groupold:blockB3 1.83 -3.42

(3.01) (3.49)

cue30cent:groupold:blockB3 -1.34 -3.75

(3.68) (4.12)

--------------------------------------------------------------------------------------------------

Scale parameter: gamma 1612.24 1577.87 1577.85 1577.82 1585.94

Scale parameter: SE 185.67 191.57 191.52 191.54 191.83

Correlation parameter: alpha 0.99 0.40 0.40 0.40 0.39

Correlation parameter: SE 0.00 0.05 0.05 0.05 0.05

Num. obs. 10954 10954 10954 10954 10954

Num. clust. 46 46 46 46 46

CorrelationStructur: AR1 EXCHNG EXCHNG EXCHNG EXCHNG

--------------------------------------------------------------------------------------------------

CIC: 910.7 63.7 59.9 56.2 10.3

QIC: 17662348 17284082 17283942 17283582 17372358

QICu: 17660563 17283991 17283850 17283490 17372349

==================================================================================================

*** p < 0.001; ** p < 0.01; * p < 0.05

Supplement table 2: GEE model overview (model parameter estimates top section, model informations middle section, model selection criteria bottom section).

M0 and M1 shows the full saturated models including the three-level factor cue, three-level factor block and 2-level

factor group, including all interactionsterms, but using different correlationstructure specifications. Based on the QIC and CIC value,

which are used to determine an suitable correlationstructur, M1 fits the data better (M0 no longer considered). M2 shows the model without

the three-way-interaction. M3 removes furthermore the interactionterm between cue and block and has the lowest QIC and QICu value, what makes M3 to the most appropiate model choice.

M4 based only on the variables cue und group und their interaction and describes the data worst under the candidate models.
