# Supplementary material for: Still Wanting to Win: Reward System Stability in Healthy Aging
Source: Front Aging Neurosci. 2022 May 30;14:863580. doi: 10.3389/fnagi.2022.863580 (PMC9190761; doi:10.3389/fnagi.2022.863580)
Supplement: Supplementary file 5 [file Table_3.docx]

Supplement table 3: cue contrasts and cue-group contrasts based on model M3 from the supplement table 2.

(SE-standarderror, LCI - lower confidence intervall, UCI - upper confidence intervall)

| contrast | group | estimate | SE | LCI | UCI | z-ratio | p-value |
| --- | --- | --- | --- | --- | --- | --- | --- |
| 0 ct - 3 ct | young | 2.819 | 0.71 | 1.43 | 4.21 | 3.970 | 0.00007 |
| 0 ct - 30 ct | young | 7.214 | 1.24 | 4.79 | 9.64 | 5.829 | 0.00000 |
| 3 ct - 30 ct | young | 4.395 | 1.14 | 2.16 | 6.63 | 3.856 | 0.00012 |
| 0 ct - 3 ct | old | 5.389 | 1.73 | 1.99 | 8.79 | 3.108 | 0.00188 |
| 0 ct - 30 ct | old | 9.376 | 2.20 | 5.06 | 13.70 | 4.253 | 0.00002 |
| 3 ct - 30 ct | old | 3.987 | 1.40 | 1.24 | 6.73 | 2.845 | 0.00444 |
| 0 ct - 3 ct | young - old | -2.570 | 1.87 | -6.24 | 1.10 | -1.372 | 0.17017 |
| 0 ct - 30 ct | young - old | -2.162 | 2.53 | -7.12 | 2.79 | -0.855 | 0.39241 |
| 3 ct - 30 ct | young - old | 0.408 | 1.81 | -3.13 | 3.95 | 0.226 | 0.82130 |
