# Supplementary material for: Still Wanting to Win: Reward System Stability in Healthy Aging
Source: Front Aging Neurosci. 2022 May 30;14:863580. doi: 10.3389/fnagi.2022.863580 (PMC9190761; doi:10.3389/fnagi.2022.863580)
Supplement: Supplementary file 6 [file Table_4.docx]

| contrast | group | estimate | SE | LCI | UCI | z-ratio | p-value |
| --- | --- | --- | --- | --- | --- | --- | --- |
| B1 - B2 | young | 4.087 | 1.22 | 1.698 | 6.476 | 3.353 | 0.00080 |
| B1 - B3 | young | 4.488 | 2.47 | -0.355 | 9.332 | 1.816 | 0.06931 |
| B2 - B3 | young | 0.401 | 1.79 | -3.098 | 3.900 | 0.225 | 0.82207 |
| B1 - B2 | old | 7.687 | 4.58 | -1.282 | 16.656 | 1.680 | 0.09299 |
| B1 - B3 | old | 17.323 | 5.89 | 5.781 | 28.864 | 2.942 | 0.00326 |
| B2 - B3 | old | 9.635 | 3.83 | 2.135 | 17.136 | 2.518 | 0.01181 |
| B1 - B2 | young - old | -3.600 | 4.74 | -12.882 | 5.682 | -0.760 | 0.44713 |
| B1 - B3 | young - old | -12.834 | 6.39 | -25.351 | -0.318 | -2.010 | 0.04446 |
| B2 - B3 | young - old | -9.234 | 4.22 | -17.511 | -0.957 | -2.187 | 0.02877 |

Supplement table 4: block contrasts and block-group contrasts based on model M3 from the supplement table 2.

(SE-standarderror, LCI - lower confidence intervall, UCI - upper confidence intervall)
